# Supplementary material for: Single cell preparations of Mycobacterium tuberculosis damage the mycobacterial envelope and disrupt macrophage interactions
Source: eLife. 2023 Feb 28;12:e85416. doi: 10.7554/eLife.85416 (PMC9998084; doi:10.7554/eLife.85416)
Supplement: Supplementary file 3. — (A) Approach used to analyze the literature to define the frequency with which distinct single cell preparation methods are used and how often they are reported. (B) Graph demonstrates the distribution of methods reported. Since some studies used multiple methods, the total does not equal 100. [file elife-85416-supp3.docx]

**
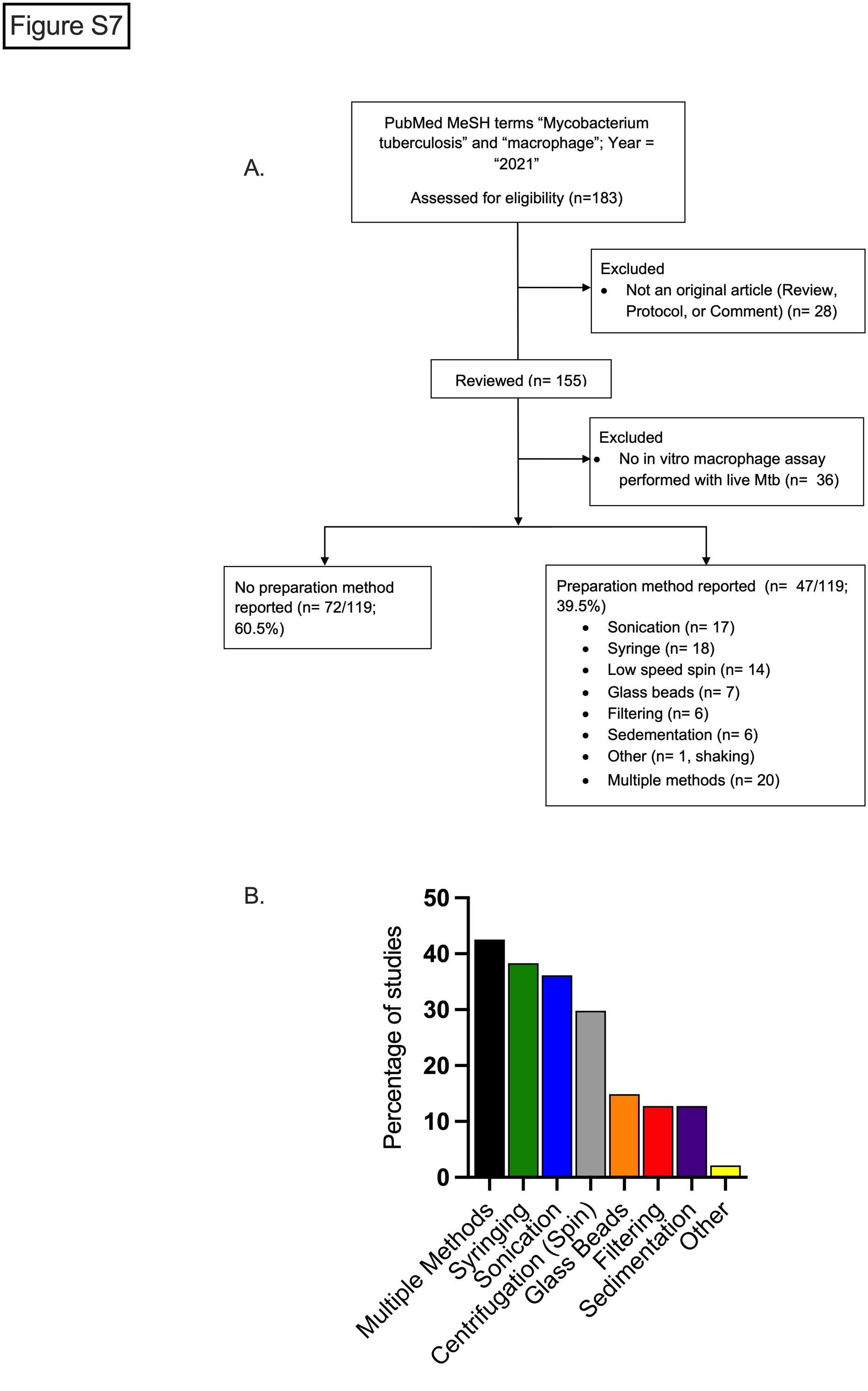
**

**Supplementary File 3. Literature review of methods used to generate single cell Mtb suspensions**

**(A)** Approach used to analyze the literature to define the frequency with which distinct single cell preparation methods are used and how often they are reported. **(B)** Graph demonstrates the distribution of methods reported. Since some studies used multiple methods, the total does not equal 100.
